# Circular RNA UVRAG Mediated by Alternative Splicing Factor NOVA1 Regulates Adhesion and Migration of Vascular Smooth Muscle Cells

**DOI:** 10.3390/genes12030418

**Published:** 2021-03-14

**Authors:** Ze Liu, Yue Lou, Jia-Chen Cui, Yi Chen, Ji-Ting Liu, Ying Yuan, Yue Han, Yun-Long Huo, Ying-Xin Qi, Zong-Lai Jiang, Qing-Ping Yao

**Affiliations:** 1 Institute of Mechanobiology& Medical Engineering, School of Life Sciences &Biotechnology, Shanghai Jiao Tong University, Shanghai 200240, China; invoker8341@sjtu.edu.cn (Z.L.); louyue327@sjtu.edu.cn (Y.L.); cuijiachen@sjtu.edu.cn (J.-C.C.); chenyiolis@sjtu.edu.cn (Y.C.); liujiting@sjtu.edu.cn (J.-T.L.); hanyue625@sjtu.edu.cn (Y.H.); huoyunlong@sjtu.edu.cn (Y.-L.H.); qiyx@sjtu.edu.cn (Y.-X.Q.); zljiang@sjtu.edu.cn (Z.-L.J.); 2 Department of Ophthalmology, Shanghai General Hospital, Shanghai Jiao Tong University School of Medicine, Shanghai 200080, China; ying.yuan@shgh.cn; 3 Key Laboratory for Biomechanics and Mechanobiology of Ministry of Education, School of Biological Science and Medical Engineering, Beihang University, Beijing 100083, China; 4 Beijing Advanced Innovation Center for Biomedical Engineering, Beihang University, Beijing 100083, China

**Keywords:** vascular smooth muscle cells, circular RNAs, vein graft, adhesion, migration, alternative splicing, NOVA1

## Abstract

The movement of abnormal vascular smooth muscle cells (VSMCs) contributes to intimal hyperplasia in vein graft disease. Circular RNAs (circRNAs) are single stranded RNAs with 3’ and 5’ ends covalently joined together. They have been shown to regulate cell function in many diseases. NOVA1 is considered to be a brain-specific splicing factor that plays an important role in the nervous system and cancer. The role of NOVA1 in VSMCs remains unclear. In the present study, transcriptome sequencing was used to identify differentially expressed circRNAs in the rat vein graft model. A novel circRNA, circUVRAG, was decreased in the grafted vein and stably located in the cytoplasm. Knockdown of circUVRAG suppressed VSMC adhesion and migration. In addition, we demonstrated that the alternative splicing factor NOVA1 co-located with UVRAG pre-mRNA in the nucleus and modulated the production of circUVRAG. These new discoveries may serve as a potential means to treat intimal hyperplasia after vein grafts.

## 1. Introduction

Myocardial ischemia caused by coronary artery obstruction is a main cause of mortality globally [1]. Despite advances in medication and percutaneous coronary intervention (PCI) technology, the coronary artery bypass graft (CABG) remains the gold standard for patients with multivessel or left main coronary artery disease [2]. Autologous veins, particularly saphenous veins, are convenient to harvest and less susceptible to vasospasm, and frequently used as donor vessels [3]. However, 2–25% of vein grafts (VGs) failed within 1 year and, by 10 years after surgery, 40–50% of VGs failed [4]. Vascular smooth muscle cells (VSMCs) proliferate and migrate to the intima, leading to intimal hyperplasia, and are an important pathological basis for vascular restenosis. Wu et al. indicated that VSMCs derived from the donor vein contributed 68% of neointimal cells in the middle segment of the graft vein [5]. Using gene therapy strategies to find specific targets that inhibit VSMCs migration to the intima may be an effective means to prevent vein graft failure. It is crucial to understand the gene expression pattern of VGs and develop new gene therapy targets.

CircRNAs form a covalently closed loop, derived from precursor mRNA usually by back-splicing due to the lack of open 3’ and 5’ ends, circRNA is resistant to exoribonuclease and more stable than its linear transcripts [6]. The functions of circRNAs in the immune system, cancer, and the nervous system have been reported [7,8,9]. In recent years, the functions of circRNAs in the cardiovascular system have received increasing attention [10]. For example, circCHFR participates in atherosclerosis by promoting the proliferation and migration of VSMCs [11]. Silencing circNfix promotes cardiomyocyte proliferation and angiogenesis [12]. Circ_Lrp6 is an intracellular regulator of miR-145 and overexpresses Circ_Lrp6 via viral delivery inhibited mouse carotid artery intimal hyperplasia [13]. The role of circRNAs in intimal hyperplasia of VGs needs further investigation.

Studies on the formation of circRNAs remain challenging. Interestingly, the same pre-mRNA produces several different circRNA variants, which indicates that alternative splicing may take part in the production of circRNAs. Some splicing factors were reported to involve in this process. Conn et al. found that the production of circRNAs was regulated by Quaking during epithelial mesenchymal transition [14]. Zhou et al. reported that epithelial-splicing regulatory protein 1 mediated the circularization of circCAMSAP1 in colorectal cancer [15]. Neuro-oncological ventral antigen 1 (NOVA1) was considered as a brain-specific splicing factor and highly expressed in the brain and other non-neural organs, such as the lung, breast, colon, and muscle [16]. In recent years, the roles of NOVA1 in neuronal cells and cancer have been revealed [17,18]. However, the function of NOVA1 has not been reported in VGs. 

In this study, we established an autologous vein graft model in rats, and used high-throughput sequencing to screen differentially expressed (DE) circRNA and mRNA. We studied the role of circUVRAG (rno-circUVRAG) in vascular remodeling. Immunofluorescence staining confirmed that NOVA1 and pre-mRNA were co-localized in the nucleus. NOVA1 regulated the production of circUVRAG and its linear transcript in VSMCs. Our studies indicate that NOVA1 is crucial for the formation of circUVRAG, and circUVRAG may be a potential therapeutic target for vein graft diseases.

## 2. Materials and Methods

### 2.1. Establishment of Vein Graft Model

Male Sprague–Dawley (SD) (280 ± 20 g) rats were purchased from Vital River Laboratory (Zhejiang, China). The feeding conditions were in accordance with the regulations of Shanghai Jiao Tong University Laboratory Animal Center. The animal experimental protocol conformed to the Animal Management Rules of China (55, 2001, Ministry of Health, China) and the study was approved by the Animal Research Committee of Shanghai Jiao Tong University.

We used the “cuff” technique to construct the VG model as previously described [19]. In brief, at an oxygen flow rate of 2 L/min, the rats were anesthetized with 2% isoflurane during the surgery. The proximal and distal ends of the left common carotid artery were clamped with vascular clips to block blood flow. Then, the vessel was cut 7 mm below the bifurcation of the left common carotid artery and a 1.2 ± 0.2 mm cannula (20 Gauge, BD, Suzhou, China) was inserted. The external jugular vein (1.5 ± 0.2 cm) on the same side was separated and placed on the cannula tying with 6.0 sutures. Finally, the neck skin was sutured with 3.0 sutures. One week after the operation, the animal was euthanized by intraperitoneal injection of excess pentobarbital sodium (75 mg/kg), and then the graft vein was harvested. At the same time, the right external jugular vein was harvested as a control.

### 2.2. High-Throughput Sequencing and Bioinformatics Analysis

High-throughput sequencing included whole transcriptome sequencing (Supplementary Materials) and miR-seq. The NovelBrain Cloud Analysis Platform (www.novelbrain.com, NovelBioinformatics Ltd., Co. accessed on 4 January 2019) was used for the bioinformatics analysis. DE genes, including mRNA, miRNA, and circRNA, in the vein graft and control were analyzed utilizing DESeq with the following criteria: fold change > 2, *p* < 0.05, and false discovery rate (FDR) < 0.05.

### 2.3. The Culture of VSMCs 

A section of the jugular vein of male SD rats was removed and cut into small pieces for culturing primary VSMCs. VSMCs were cultured in Dulbecco’s modified Eagle medium (DMEM, Gibco, Waltham, MA, USA) containing 10% fetal bovine serum (FBS, Gibco) and 1% penicillin/streptomycin solution (BBI Life Science, Shanghai, China) in a humidified incubator at 37 °C and 5% CO2. The purity of VSMCs was detected by immunofluorescence staining using specific marker smooth muscle α-actin (SMA) (Proteintech, Chicago, IL, USA). VSMCs between 4 and 7 passages and purity more than 95% were used for all experiments.

### 2.4. Immunofluorescence and Fluorescence In Situ Hybridization (FISH)

For FISH, VSMCs were washed with 1× PBS solution three times at room temperature. The cells were fixed with 4% paraformaldehyde and 0.3% Triton X-100 in PBS mixture for 30 min and then washed with PBS. Pre-hybridization buffer was added at 37 °C for 30 min. Hybridization was carried out with a FISH probe in a moist chamber at 37 °C in the dark overnight using a Ribo FISH Kit (C10910, RiboBio, Guangzhou, China). The cells were washed with hybridization washing buffer at 42 °C. The nuclei were stained with 4′,6-diamidino-2-phenylindole (DAPI) (Sigma-Aldrich, St. Louis, MO, USA). For immunofluorescence, after two steps of fixing and permeabilization, the cells were blocked with 10% goat serum and then incubated with primary antibody at 4 °C overnight. The cells were washed with PBS and then incubated with Alexa Fluor 488 (1:500, anti-mouse, Cell Signaling Technology, Danvers, MA, USA) at room temperature for 2 h. The cellular location of NOVA1 was identified by immunofluorescence staining with NOVA1 antibody (1:100, novus). FISH was photographed by laser scanning confocal microscope (Olympus, FLUOVIEW FV1000) and the co-localization assay was photographed by Leica TCS SP8 STED. 

### 2.5. RNase R Digestion and Actinomycin D Treatment

Ribonuclease R (RNase R, Epicentre, Madison, WI, USA) and actinomycin D (MedChemExpress, South Brunswick, NJ, USA) were used to evaluate the stability and half-life of circular RNA. A quantity of 1 ug total RNAs was digested with 3U RNase R at 37 °C for 10 min. After stopping the reaction at 70 °C for 10 min, circRNA and linear mRNA were detected by qPCR. Samples without RNase R treatment were used as the control. Actinomycin D (500 ng/mL) or DMSO (Sangon Biotech, Shanghai, China) was added to DMEM medium of VSMCs. After 24 h, circRNA and linear mRNA was detected by qPCR.

### 2.6. Cell Adhesion and Migration

After transfecting the cells with siRNA for 48 h, VSMCs were digested and reseeded in a 24-well plate. After 15 min, the unadhered VSMCs were washed away with PBS. Through fixing and permeabilizing, adherent cells were stained with DAPI. Photos were taken under a microscope (Olympus, IX-71, Tokyo, Japan) to count the number of adherent cells. For the wound healing assay, VSMCs were seeded into 6-well plates. Using a 200-mL pipette tip, the monolayer of cells was gently scratched to create a straight line when the cells reached a 90% confluence. Photographs were taken after scratching for 24 h. Image J was used to calculate cell migration area (the difference between the scratch area at the beginning (0 h) of the experiment and 24 h later). For the transwell assay, VSMCs were digested with trypsin and resuspend in DMEM. A quantity of 40 μL cell suspension (1 × 10^4^ cells) was seeded into a transwell insert (CORNING, Corning, NY, USA). The chamber was placed in a 6-well plate which was added 1 mL of DMEM medium containing 10% FBS. After 8 h, paraformaldehyde was used to fix cells for 20 min and VSMCs were treated with 0.3% Triton-100 for 5 min. Using a cotton bar, cells were wiped off in the top of transwell insert after staining cells with hematoxylin. The remaining cells were photographed under the microscope and calculated with Image J. 

### 2.7. SiRNA Transfection

Small interfering RNA (siRNA) targeting circUVRAG was designed and synthesized by Gene Pharma (Shanghai, China). Briefly, 1 × 10^5^ cells were seeded into 6-well plates, and then VSMCs were transfected with 100 nM siRNA fragments with Lipofectamine 2000 reagent (Invitrogen, Waltham, MA, USA) and Opti MEM (Invitrogen). After 48 h, cells were lysed with TRIzol reagent (Invitrogen) for RNA extraction. The siRNA fragment sequences are listed in Supplementary Table S1.

### 2.8. RNA Extraction and qPCR

Total RNA was extracted with TRIzol reagent. Spectrophotometry (TIANGEN, OSE-260, Beijing, China) was used to detect the concentration and purity of RNA. A quantity of 1 μg total RNA was used as a template for reverse transcription PCR to synthesize complementary DNA (cDNA). Quantitative PCR (qPCR) was performed on a Step One plus PCR system (Thermo Fisher Scientific, Waltham, MA, USA) with SYBR green Premix Ex Taq (TakaRa, Kyoto, Japan). The sequences of primers are shown in Supplementary Table S1. 

### 2.9. Statistical Analysis

All data are shown as the mean ± SEM. Each statistical data experiment was repeated at least three times. Prism 8 (GraphPad, San Diego, CA, USA) was used for statistical calculations. Student’s *t*-test was used to analyze the comparison between two groups and one-way ANOVA was used for comparison among three or more groups. *p*-value < 0.05 was considered to be statistically significant.

## 3. Results

### 3.1. Identification and Characterization of Differentially Expressed Circular RNA

**Figure 1:**
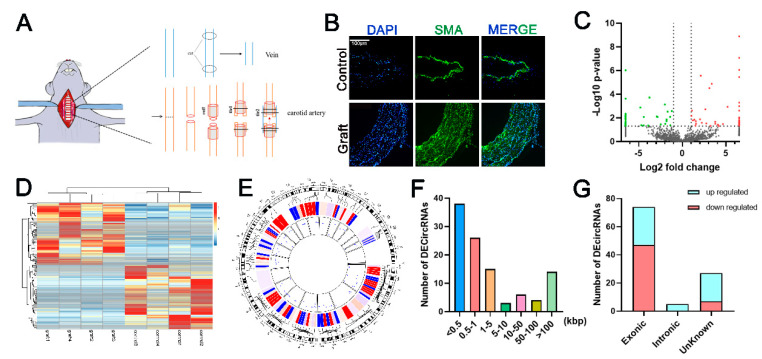
Characterization of differentially expressed circular RNA. (**A**). Schema of the rat vein graft model using “cuff” technique. One week after surgery, graft vein and the right jugular vein were harvested for the subsequent experiments, *n* = 4. (**B**). Immunofluorescent staining shows that the vascular wall of graft vein is thicker than those in the control group. 4′,6-diamidino-2-phenylindole (DAPI) stains the nucleus blue, and green represents vascular smooth muscle cells (VSMC)-specific marker smooth muscle α-actin. Bar = 100 μm. (**C**). Volcano map of circular RNA (circRNA) expression. Two vertical dotted lines correspond to the fold change of ±2.0. The horizontal dotted line represents that *p* = 0.05. The red dots and green dots indicate the significantly increased and decreased circRNAs in the graft vein, respectively. (**D**). Heat map shows 106 DE circRNAs. DE CircRNAs are clustered by https://biit.cs.ut.ee/clustvis/ (accessed on 7 September 2020). (**E**). Circlize represents the distribution of DE circRNAs on rat chromosomes. The red lines present upregulated and the blue lines are downregulated circRNAs. (**F**). DE circRNAs length distribution. (**G**). DE circRNAs category distribution.

An in vivo VG model was established by the “cuff” technique in rats (Figure 1A). The graft vein and self-contralateral jugular vein were collected one week after the VG operation for follow-up experiments. Smooth muscle α-actin (SMA, a VSMC specific marker) was dyed green and DAPI was used to stain the nucleus blue. Immunofluorescent staining for SMA showed that the vascular wall of the graft vein was thicker than that in the control group and VSMCs contributed to the thickening of the vascular wall (Figure 1B). Four groups of VGs and the control veins were obtained for RNA sequencing. As shown in the volcano figure (Figure 1C), whole-transcriptome sequencing revealed that 2048 circRNAs were identified and 106 DE circRNAs were filtrated using *p* < 0.05 and fold change > 2 as the criteria. The heat map showed that 54 circRNAs were up-regulated and 52 were down-regulated (Figure 1D). DE circRNAs were distributed on all chromosomes except the 12th, 18th, and sex chromosomes (Figure 1E). The length of most DE circRNAs was within 5000 bp (Figure 1F). Most of the DE circRNAs were derived from exon circularization. Compared to the control, 36.49% of exon circRNAs were up-regulated and 63.51% were down-regulated in the VG group (Figure 1G). 

### 3.2. The Expression and Location of circUVRAG in VSMCs

**Figure 2:**
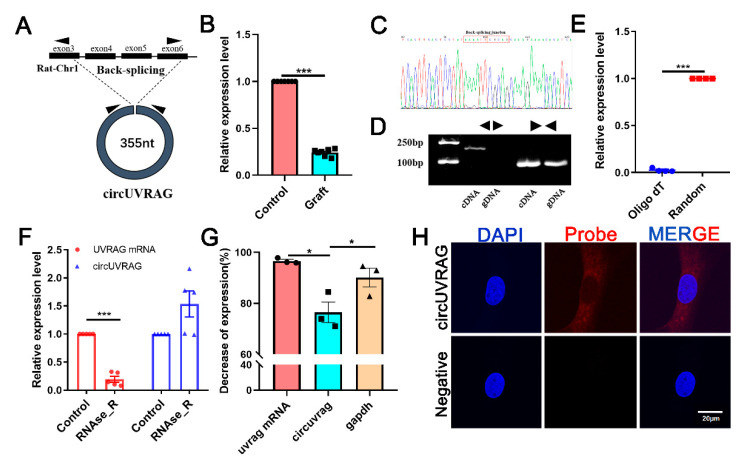
Identification and location of circUVRAG in rat VSMCs. (**A**). Schema shows that circUVRAG is derived from exons 3, 4, 5 and 6 of *uvrag*. Divergent primers are designed to detect circUVRAG. (**B**). QPCR confirms that circUVRAG is decreased in the graft vein compared to the control (*n* = 7). (**C**). Sanger sequencing demonstrates the back-splicing junction of circUVRAG by using divergent primers. (**D**). PCR and agarose gel electrophoresis show that divergent primers amplify circUVRAG in cDNA but not gDNA. (**E**). The amplification efficiency with oligo (dT) primer is much lower than random primer (*n* = 4). (**F**). With RNase R treatment, linear UVRAG mRNA is digested and circUVRAG shows higher stability (*n* = 5). (**G**). Actinomycin D is utilized to block the transcription of DNA for 24 h. The consumption of circUVRAG is much less than linear UVRAG mRNA and GAPDH (*n* = 3). (**H**). Fluorescence in situ hybridization (FISH) assay shows that circUVRAG is mainly located in the cytoplasm of VSMCs. DAPI stains the nucleus blue and red indicates circUVRAG. Bar = 20 μm, * *p* < 0.05, *** *p* < 0.001.

A novel circRNA chr1_164064815_164025518_-39297-UVRAG (circUVRAG) was screened considering the sequence length, *p*-value, and fold change. The expression of circUVRAG was decreased in the graft vein compared to the control. CircUVRAG was formed by the back splicing of exons 3, 4, 5, and 6 of the *Uvrag* gene located on Rattus norvegicus chromosome 1 with a sequence length of 355 bp (Figure 2A). QPCR results verified that the circUVRAG expression in the graft vein was significantly decreased compared to the control group (Figure 2B). Sanger sequencing validated the head-to-tail junction of circUVRAG using divergent primers (Figure 2C). To further confirm the circular structure, convergent and divergent primers were designed to amplify complementary DNA (cDNA) and genomic DNA (gDNA). As shown in Figure 2D, using both cDNA and gDNA as a template, there were PCR products with the convergent primer; with the divergent primer, there were no PCR products via using gDNA as a template. This suggests that circularization of circUVRAG needed transcription. Moreover, because circUVRAG does not have a 3’poly A tail, the amplification efficiency with the oligo (dT) primer was much lower than that of the random primer (Figure 2E). With RNase R treatment, linear UVRAG mRNA was digested. However, the expression of circUVRAG did not decrease, which indicates that circRNA can resist digestion by RNase R due to its unique circular structure (Figure 2F). In addition, after blocking transcription of DNA for 24 h with actinomycin D, circUVRAG had higher stability than linear UVRAG mRNA and glyceraldehyde 3-phosphate dehydrogenase (GAPDH) (Figure 2G). The above results firmly confirmed the circular structure of circUVRAG. The fluorescence in situ hybridization assay showed that circUVRAG was mainly located in the cytoplasm of VSMCs (Figure 2H).

### 3.3. Knockdown of circUVRAG Inhibits VSMCs Adhesion and Migration

**Figure 3:**
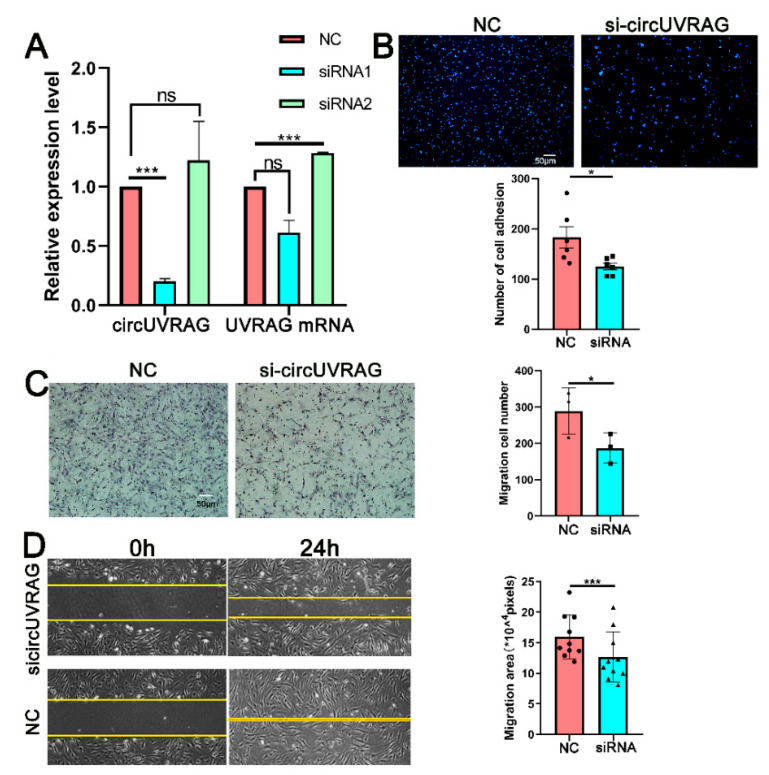
Knockdown of circUVRAG inhibits VSMC adhesion and migration. (**A**). Two small interference RNA (siRNA) sequences targeting the back-splicing junction are designed to specifically reduce circUVRAG. SiRNA1 effectively reduced the expression of circUVRAG by 70% without affecting the expression of linear UVRAG mRNA (*n* = 3). (**B**). Knockdown of circUVRAG inhibits VSMC adhesion (*n* = 6). (**C**,**D**). Transwell assay (*n* = 3) (**C**) and wound healing assay (*n* = 10) (**D**) demonstrate that knockdown of circUVRAG inhibits VSMC migration. Bar = 50 μm, * *p* < 0.05, *** *p* < 0.001.

To explore the function of circUVRAG, two small siRNA sequences targeting the back-splicing junction were designed to specifically reduce circUVRAG. The qPCR results showed that siRNA1 effectively reduced the expression of circUVRAG by 70% without affecting the expression of linear UVRAG mRNA (Figure 3A). In subsequent experiments, we used siRNA1 to knockdown the expression of circUVRAG. Intimal hyperplasia is closely related to the movement of VSMCs, so we detected the VSMC adhesion and migration after transfecting circUVRAG-specific siRNA1(si-circUVRAG). There was less VSMC adhesion compared to the transfecting of negative control fragments (NC). DAPI staining and statistical results are shown in Figure 3B. Transwell assay was performed to evaluate the invasion ability of VSMCs with hematoxylin staining. Figure 3C shows that the number of cells across the 8 μm insert was decreased significantly after interfering with circUVRAG. In addition, Figure 3D shows that knockdown of circUVRAG expression significantly decreased cell migration detected by wound healing assay. These results indicate that knockdown of circUVRAG decreased the VSMC adhesion to the matrix and significantly reduced cell migration.

### 3.4. The Splicing Factor NOVA1 Co-Localizes with UVRAG pre-mRNA in the Nucleus

**Figure 4:**
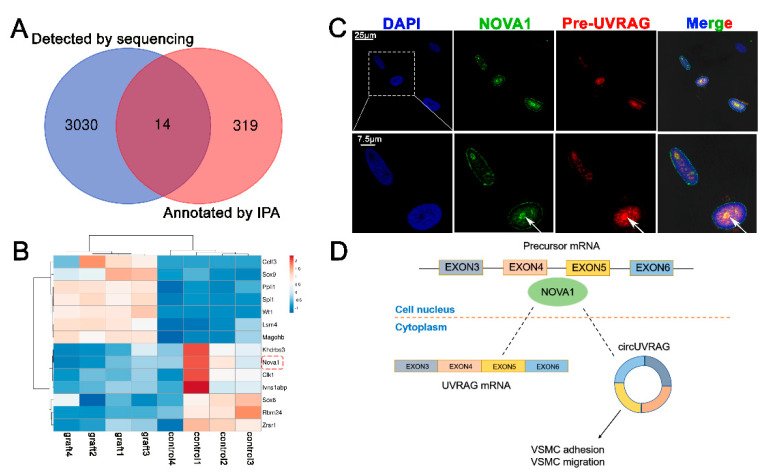
The splicing factor NOVA1 co-localizes with UVRAG pre-mRNA in the nucleus. (**A**). Intersecting the 3044 differentially expressed (DE) genes obtained by whole-transcriptome sequencing with the 333 genes related to alternative splicing annotated by the Ingenuity Pathway Analysis (IPA) database, we identified 14 DE alternative splicing factors. (**B**). The heat map shows that NOVA1 is decreased in the graft vein. (**C**). Immunofluorescence and fluorescence in situ hybridization assays indicate that NOVA1 protein and UVRAG pre-mRNA co-localize in the nucleus. NOVA1 protein is green and UVRAG pre-mRNA is red. The two types of fluorescence at the position indicates by the white arrows are superimposed in yellow (Bar = 25 μm, and 7.5 μm). (**D**). NOVA1 might be involved in the formation of circUVRAG in VSMCs.

Circular RNA was formed by a special splicing mechanism. Our sequencing results predicted abundant alternative splicing events in VGs (Supplementary Figure S1). After confirming that circUVRAG was a functional circRNA, we wondered if a splicing factor was involved in its formation. Intersecting the 3044 DE genes obtained by whole-transcriptome sequencing with the 333 genes related to alternative splicing annotated by the Ingenuity Pathway Analysis (IPA) (https://digitalinsights.qiagen.com/products-overview/discovery-insights-portfolio/analysis-and-visualization/qiagen-ipa/, version No: 39480507; accessed on 19 October 2020), we identified 14 DE alternative splicing factors (Figure 4A). The heat map shows the list of 14 DE alternative splicing factors. NOVA1, a brain-specific alternative splicing factor, has a considerable level of expression in muscles, whereas its function in VSMCs has not been reported. The expression of NOVA1 decreased in the graft vein (Figure 4B). According to the predictions based on the structure of RNA and protein binding (http://pridb.gdcb.iastate.edu/RPISeq/, and http://bioinfo.bjmu.edu.cn/lncpro/; accessed on 23 November 2020), the NOVA1 protein may bind to the circUVRAG (Table 1). 

Pre-mRNA processes in the nucleus and NOVA1 protein are also mainly located in the nucleus as reported. In VSMCs, we performed both NOVA1 immunofluorescence (green) and UVRAG pre-mRNA (red) fluorescence in situ hybridization assays. The results showed that NOVA1 protein and UVRAG pre-mRNA co-localized in the nucleus. The two types of fluorescence at the position indicated by the white arrow are superimposed in yellow. These results suggest that NOVA1 might be involved in alternative splicing of UVRAG pre-mRNA in VSMCs (Figure 4C) and may affect the formation of circUVRAG in VSMCs (Figure 4D). 

### 3.5. Silencing NOVA1 Reduced the Expression of circUVRAG and Linear UVRAG mRNA without Affecting the Expression of UVRAG pre-mRNA

**Figure 5:**
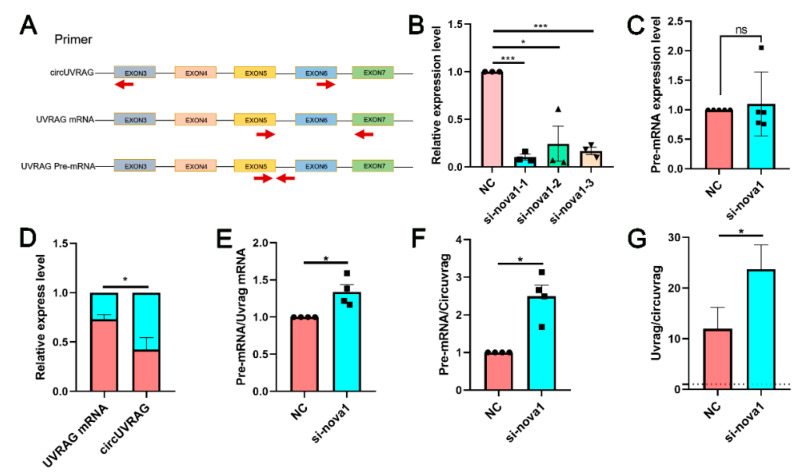
Silencing NOVA1 reduces the expression of circUVRAG and linear UVRAG mRNA without affecting the expression of UVRAG pre-mRNA. (**A**). The schema of primer design for circUVRAG, UVRAG mRNA, and UVRAG pre-mRNA. (**B**). Three siRNA fragments are designed to reduce the expression of NOVA1 mRNA. Among these, siRNA1 is the most efficient and stable (*n* = 3). (**C**). Knockdown of NOVA1 has no effect on the abundance of UVRAG pre-mRNA (*n* = 5). (**D**). Both circUVRAG and UVRAG mRNA decrease after knocking down NOVA1. CircUVRAG is reduced significantly more than UVRAG mRNA (*n* = 5). (**E**–**G**). The ratio of pre-mRNA to UVRAG mRNA (**E**) (*n* = 4) and circUVRAG (**F**) (*n* = 4) increases and the ratio of linear UVRAG mRNA to circUVRAG (**G**) (*n* = 7) also increases after reducing NOVA1. * *p* < 0.05, *** *p* < 0.001.

To investigate the relationship of NOVA1 and circUVRAG, we reduced the expression of NOVA1 and circUVRAG separately. After knockdown of circUVRAG, the expression of NOVA1 mRNA did not change significantly (Supplementary Figure S2), which indicates that NOVA1 was not regulated by circUVRAG. Figure 5A shows the schema of primer design for circUVRAG, UVRAG mRNA, and UVRAG pre-mRNA. To explore whether NOVA1 affects the production of circUVRAG, three siRNA fragments specifically targeting NOVA1 mRNA were designed. The qPCR results showed that three siRNA fragments effectively reduced the expression level of NOVA1 mRNA. Among these, siRNA1 was most efficient (Figure 5B) and siRNA1 was used to interfere with the expression of NOVA1 in subsequent experiments. The siRNA1 of NOVA1 did not affect the abundance of UVRAG pre-mRNA (Figure 5C). Both circUVRAG and UVRAG mRNA expression were reduced after transfection of siRNA1. However, circUVRAG was reduced significantly more than UVRAG mRNA, which suggested that NOVA1 was crucial to the formation of circUVRAG (Figure 5D). The ratio of pre-mRNA to UVRAG mRNA and circUVRAG increased and the ratio of linear UVRAG mRNA to circUVRAG also increased (Figure 5E–G). In summary, after reducing the expression of NOVA1, the splicing process of UVRAG pre-mRNA was inhibited. The formation of circUVRAG and mature UVRAG mRNA was blocked. NOVA1 played an important role in regulating UVRAG pre-mRNA splicing in VSMCs and affected the formation of circUVRAG.

## 4. Discussion

Intimal hyperplasia caused by abnormal proliferation and migration of VSMCs is a major cause of restenosis after VGs [20]. Exploring the molecular therapeutic targets that affect VSMC function is a good strategy to prevent surgical failure caused by excessive intimal hyperplasia. Based on high-throughput sequencing, we screened circUVRAG and focused on the effect of circUVRAG on the adhesion and migration of VSMCs, and furthermore explored the mechanism of circUVRAG formation.

Many studies believe that the excessive migration of VSMCs to the intima causes vascular stenosis. Cell adhesion and migration are highly correlated. If cell adhesion to the matrix is too stable, the cells fail to migrate effectively. On the contrary, unstable adhesion may weaken the connection of cells to the matrix and the generation of traction force, thereby affecting cell migration [21]. Our bioinformatic analysis revealed several DE genes in graft veins, such as itga7, mylk, ppp1r12a, ppp1r12b, and igf1r. These genes are related to the focal adhesion pathway, and may be the downstream targets of circUVRAG, which warrant further investigation. CircRNAs have received increasing attention as therapeutic targets for intimal hyperplasia. Our previous research showed that knockdown of circtet3 inhibited the migration of VSMCs [22]. Research by Rong et al. confirmed that knockdown of circDcbld1 in vivo reduced the formation of neointima in the rat common carotid artery after balloon injury [23]. Specific transfection of CircMap3k5 in vivo inhibited the proliferation of VSMCs in the arteries of mice, resulting in a decrease in neointima formation [24]. Sun et al. indicated that abnormal expression of circACTA2 and miR-548f-5p was involved in intimal hyperplasia [25]. In the present study, our results demonstrated that knockdown of circUVRAG in vitro repressed VSMC adhesion and migration. This is consistent with the results obtained by Yang et al. in exploring the role of circUVRAG in bladder cancer [26]. However, knockdown of circUVRAG had no significant effect on the VSMC proliferation detected with the BrdU assay (Supplementary Figure S3). The reduced circUVRAG may play a role in protecting the vessels from excessive thickening. According to the circBase database (http://circbase.org/; accessed on 15 January 2021), circUVRAG is highly conserved in humans, rats, and mice. Supplementary Table S2 lists the homologous circUVRAG in humans and mice and Supplementary Figure S4 shows the sequence of circUVRAG. CircUVRAG has the potential to be a molecular therapeutic target for vein graft disease.

The mechanism that circUVRAG regulates cell adhesion and migration remains to be further studied. To date, most research has concentrated on the function of circRNAs in binding microRNAs [27,28]. Our whole transcriptome sequencing predicted that four miRNAs, namely, rno-miR-3551-5p, rno-miR-666-5p, rno-miR-345-5p, and rno-miR-93-3p, may combine with circUVRAG. However, to be a miRNA sponge, circRNA is supposed to have rich miRNA binding sites and considerable expression abundance. The length of circUVRAG was only 355 bp, which may limit its ability to adsorb miRNAs. Additionally, some circRNAs interacted with RNA binding proteins to work as scaffolds to mediate the formation of the protein complex [29,30]. In the light of the prediction of the website (http://service.tartaglialab.com/page/catrapid_group; accessed on 23 November 2020), circUVRAG may also execute a function by binding protein (Supplementary Table S3). Furthermore, circRNAs may affect cell destiny by influencing the expression of its host gene. Wu et al. found that circYAP reduced the expression of YAP protein by inhibiting the assembly of the Yap translation initiation site [31]. Li et al. indicated that circITGA7 promoted the transcription of linear ITGA7 through the Ras pathway [32]. Kong et al. confirmed that circ-Sirt1 promoted the expression of the host gene SIRT1 by binding miR-132/212 [33]. Exploring the relationship between circUVRAG and UVRAG mRNA may be worthy of future work. 

In this article, we preliminarily studied the effect of splicing factors on the formation of circRNAs and the expression levels of circRNAs and linear transcripts. Some research reported that flanking intron sequence pairing and RNA binding protein (RBP) docking on specific motifs promote circRNA formation. In addition, the expression of most circRNAs was significantly lower than that of linear transcripts (less than 10% of related linear mRNAs) [34]. Consistent with this report, qPCR results showed that the expression level of UVRAG mRNA was more than 10 times that of circUVRAG (Figure 5G). Moreover, 20 isoforms of has-circUVRAG and 3 isoforms of mmu-circUVRAG are included in the circBase database, which might due to alternative splicing. Licht et al. confirmed that the lack of NOVA1 modulated RNA editing [35]. In present study, NOVA1 has been for the first time detected in rat VSMCs in which it is located in the nucleus. However, the circUVRAG is located in cytoplasm. Immunofluorescence staining showed that NOVA1 co-localized with UVRAG pre-mRNA in nucleus. Therefore, NOVA1 may attend processing UVRAG pre-mRNA and modulating circUVRAG formation. Our results showed that when NOVA1 was impeded, the expression of circUVRAG and UVRAG mRNA was reduced and the production of circUVRAG underwent a greater decline. The alternative splicing might involve the formation of circRNAs and then modulate VSMC function.

## 5. Conclusions

In summary, we discovered a functional circular RNA, circUVRAG, that inhibited the adhesion and migration of VSMCs. The alternative splicing factor NOVA1 is involved in the process of circUVRAG formation. These results provide a new perspective for the intervention of restenosis after vein grafts.

## Figures and Tables

**Table 1:** The NOVA1 protein may bind to the circUVRAG.

CircRNA	Protein	RPIseq		lncPro
		RF	SVM	Value
CircUVRAG	NOVA1	0.75	0.85	66.5614
CircUVRAG	CELF3	0.8	0.93	44.1834
CircUVRAG	CLK1	0.75	0.91	63.512
CircUVRAG	IVNS1ABP	0.75	0.78	41.1168
CircUVRAG	KHDRBS3	0.8	0.93	34.8158
CircUVRAG	LSM4	0.75	0.862	31.5662
CircUVRAG	MAGOHB	0.75	0.677	34.8675
CircUVRAG	PPIL1	0.8	0.851	54.9877
CircUVRAG	RBM24	0.65	0.768	57.5284
CircUVRAG	SOX6	0.75	0.94	58.9258
CircUVRAG	SOX9	0.75	0.888	49.6005
CircUVRAG	SPI1	0.65	0.843	32.2629
CircUVRAG	WT1	0.7	0.91	48.7542
CircUVRAG	ZRSR1	0.75	0.926	57.8013

## Data Availability

Not available.

## Supplementary Materials

The following are available online at https://www.mdpi.com/2073-4425/12/3/418/s1, Figure S1: Our sequencing results predicted abundant alternative mRNA splicing events in graft vein. Cassette: cassette exon, i.e., Skipped exon; Cassette_multi: multiple adjacent Cassette exons; A5SS: Alternative 5’ splice site; A3SS: Alternative 3’ splice site; AltStart: Alternative start exon; AltEnd: Alternative end exon; MXE: Mutually exclusive exons; IR: intron retention, Figure S2: After knockdown of circUVRAG, the expression of NOVA1 mRNA did not change significantly, which indicated that NOVA1 was not regulated by circUVRAG (*n* = 3), Figure S3: Knockdown of circUVRAG had no significant effect on the VSMC proliferation detected with the BrdU assay (*n* = 4). A colorimetric BrdU kit (Roche Diagnostics) was used to detect the proliferation of VSMCs. An enzyme linked immunosorbent assay plate reader (Bio-Rad 680) was used to measure the absorbance of culture medium with a wavelength of 450 nm and 630 nm, Figure S4: The sequence of circUVRAG in rat. Table S1: PCR primer sequence, siRNA sequence, and FISH probe sequence, Table S2: The homologous circUVRAG in humans and mice. Table S3: The top 10 RNA Binding Proteins predicted by catrapid.

## Abbreviations

CABG	Coronary artery bypass graft
cDNA	Complementary DNA
DAPI	4′,6-diamidino-2-phenylindole
DE circRNA	Differentially expressed circRNA
DE mRNA	Differentially expressed mRNA
FBS	Fetal bovine serum
FDR	False discovery rate
FISH	Fluorescence in situ hybridization
GAPDH	Glyceraldehyde 3-phosphate dehydrogenase
gDNA	Genomic DNA
IPA	Ingenuity Pathway Analysis
NC	Negative control
NOVA1	Neuro-oncological ventral antigen 1
PCI	Percutaneous transluminal coronary intervention
qPCR	Quantitative polymerase chain reaction
RBP	RNA binding protein
SMA	Smooth muscle α-actin
VGs	Vein grafts
VSMCs	Vascular smooth muscle cells
