# Supplementary material for: Circular RNA UVRAG Mediated by Alternative Splicing Factor NOVA1 Regulates Adhesion and Migration of Vascular Smooth Muscle Cells
Source: Genes (Basel). 2021 Mar 14;12(3):418. doi: 10.3390/genes12030418 (PMC7999860; doi:10.3390/genes12030418)
Supplement: Supplementary file 1 [file genes-12-00418-s001.pdf]

**Supplementary Materials:**

**Method of transcriptome sequencing**

4 pairs of graft veins and control were obtained after one week of surgery. RNA from samples extracted by Trizol reagent (Invitrogen) with RIN>6.0 was utilized to construct rRNA depletion library (NEBNext Ultra Directional RNA Library Prep Kit) according to the manufacturer's instructions. Whole transcriptome sequencing data sequenced by HiseqTM Sequencer was filtered (removing the adaptor sequences, reads with >5% ambiguous bases (noted as N) and low-quality reads containing more than 20 percent of bases with qualities of <20) and mapped to rat genome (Btagenome Version 5.0.1 NCBI) utilizing HISAT2 was used to calculate the gene count of mRNA and non-coding RNAs. NovelBrain Cloud Analysis Platform (www.novelbrain.com, NovelBioinformatics Ltd., Co.) was used for the bioinformatics analysis. DE genes, including mRNA, miRNA, and circRNA, in the vein graft and control were analyzed utilizing DESeq with the following criteria: fold change > 2, $p < 0.05$, and false discovery rate (FDR) < 0.05.

Supplementary Fig. S1. Our sequencing results predicted abundant alternative mRNA splicing events in graft vein. Cassette: cassette exon, i.e. Skipped exon; Cassette_multi: multiple adjacent Cassette exons; A5SS: Alternative 5' splice site; A3SS: Alternative 3' splice site; AltStart: Alternative start exon; AltEnd: Alternative end exon; MXE: Mutually exclusive exons; IR: intron retention

Supplementary Fig. S2. After knockdown of circUVRAG, the expression of NOVA1 mRNA did not change significantly, which indicated that NOVA1 was not regulated by circUVRAG(n=3).

Supplementary Fig. S3. Knockdown of circUVRAG had no significant effect on the VSMC proliferation detected with the BrdU assay (n=4). Colorimetric BrdU kit (Roche Diagnostics) was used to detect the proliferation of VSMCs. Enzyme linked immunosorbent assay plate reader (Bio-Rad 680) was used to measure the absorbance of culture medium with a wave length of 450 nm and 630 nm.

Supplementary Fig. S4. The sequence of circUVRAG in rat.

**S1**

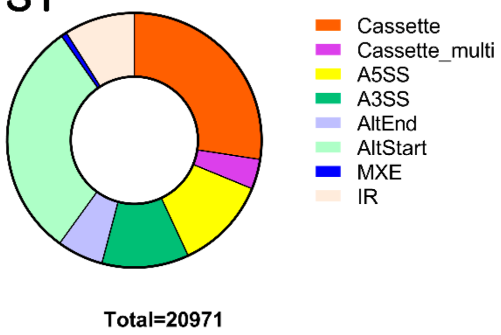


**S2**

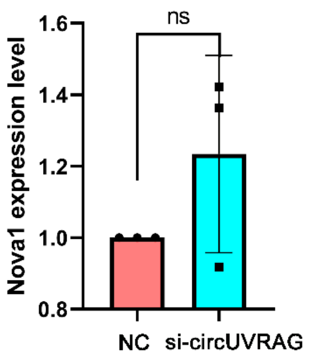


**S3**

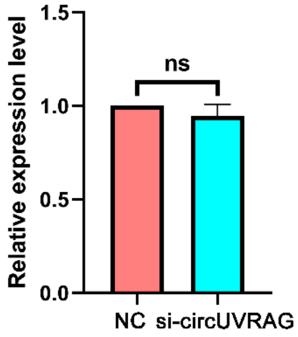


**S4**

>chr1_164064815_164025518_-39297-Uvrag
AATTTTATAGGAGTGAAGTGATTAAGAATTC
CTTGAATCCAACGTGGAGGAGTCTTGACT
TTGGAATAATGCCGGACCGTCTGGATACAT
CTGTGTCCTGCTTTGTGGTGAAGATTTGGG
GTGGAAAGGAAGAGGCCTATCAGCTGTTGA
TAGAGTGGAAAGTCTACTTGGATGGGCTGA
AGTACTTGGGTCAGCAGATCCATGCCCGCA
ACCAGAATGAAATCATTTTTGGGCTGAACG
ATGGCTACTACGGTGCTCCATTTGAACACA
AGGCTCATCCAAATGCACAGAAGAACCTCC
TTCAGGTGGACCAGAACTGTGTTCGCAATT
CCTATGATGTGTTTTCTTTGCTGCG

**Supplementary Table S1.** PCR primer sequence, siRNA sequence, and FISH probe sequence

| PCR primer sequence, siRNA sequence, and FISH probe sequence | | |
|---|---|---|
| **Primer sequence** | | |
| circUVRAG | Forward | TGGACCAGAACTGTGTTCGCA |
| | Reverse | GCCTCTTCCTTTCCACCCCA |
| uvrag-mRNA | Forward | GGCTACTACGGTGCTCCATT |
| | Reverse | CATTGCTTGTCGATGTGAGTCT |
| uvrag-premRNA | Forward | GGCTGAACGATGGCTACTACG |
| | Reverse | CCGACTTCTCAGAGTGCTTGTG |
| uvrag for gDNA | Forward | GGAATAATGCCGGACCGTCT |
| | Reverse | ACAGCTGATAGGCCTCTTCCT |
| nova1 | Forward | GCAGCACCAAGAGGACCAACAC |
| | Reverse | CTTGATCGTGGCTCCCGTTTCC |
| gapdh | Forward | TGAACTTGCCGTGGGTAGAG |
| | Reverse | GATGGTGAAGGTCGGTGTGA |
| **siRNA sequence** | | |
| Negative control | Forward | UUCUUCGAACGUGUCACGUTT |
| | Reverse | ACGUGACACGUUCGGAGAATT |
| siRNA1 for circUVRAG | Forward | UUCUUUGCUGCGAAUUUUATT |
| | Reverse | UAAAAUUCGCAGCAAAGAATT |
| siRNA2 for circUVRAG | Forward | UUGCUGCGAAUUUUAUAGGTT |
| | Reverse | CCUAUAAAAUUCGCAGCAATT |

| | | |
|---|---|---|
| siRNA1 for nova1 | Forward | GCUGCUCAGUAUUUAAUUATT |
| | Reverse | UAAUUAAAUACUGAGCAGCTT |
| siRNA2 for nova1 | Forward | GGUCCUCAUACCUAGUUAUTT |
| | Reverse | AUAACUAGGUAUGAGGACCTT |
| siRNA3 for nova1 | Forward | GCUGCAACCAAUGGAUACUTT |
| | Reverse | AGUAUCCAUUGGUUGCAGCTT |
| **Fish probe sequence** | | |
| Negative control | sense（5'-3'） | TGCTTTGCACGGTAACGCCTGTTTT |
| circUVRAG probe mix | sense（5'-3'） | GGCCCCAAATCAGAGTCCAT |
| | sense（5'-3'） | CCGGGGAAGGAAGGGAAGAG |
| | sense（5'-3'） | CCCCAAATCAGAGTCCATGG |

**Supplementary Table S2.** The homologous circUVRAG in humans and mice

| Position (genome browser link) | Strand | circRNA ID | Genomic length | Spliced length | Best transcript |
|---|---|---|---|---|---|
| chr7:106247821-106266815 | - | mmu_circ_0013695 | 18994 | 315 | ENSMUST00000037968 |
| chr7:106214309-106247983 | - | mmu_circ_0001601 | 33674 | 320 | NM_178635 |
| chr11:75572790-75672593 | + | hsa_circ_0023635 | 99803 | 464 | NM_003369 |
| chr11:75572790-75623083 | + | hsa_circ_0023634 | 50293 | 358 | NM_003369 |
| chr11:75562927-75694557 | + | hsa_circ_0023633 | 131630 | 709 | NM_003369 |
| chr11:75562927-75623083 | + | hsa_circ_0096459 | 60156 | 476 | NM_003369 |
| chr11:75562927-75599947 | + | hsa_circ_0140972 | 37020 | 390 | NM_003369 |
| chr11:75562927-75599947 | + | hsa_circ_0096458 | 37020 | 390 | NM_003369 |

**Supplementary Table S3.** The top 10 RNA Binding Proteins predicted by catrapid.

| Protein | Transcript | Z-score | Discriminative Power | Interaction Strength | Motif |
|---|---|---|---|---|---|
| sp\|P63155\|CRNL1_RAT | chr1_164064815__1 | 0.91 | 0.96 | 0.88 | NO |
| sp\|Q496Z9\|TRM1L_RAT | chr1_164064815__1 | 0.81 | 0.96 | 0.8 | NO |
| sp\|Q99PF5\|FUBP2_RAT | chr1_164064815__1 | 0.78 | 0.95 | 0.79 | NO |
| sp\|Q641Y8\|DDX1_RAT | chr1_164064815__1 | 0.71 | 0.95 | 0.85 | NO |
| sp\|Q7TP47\|HNRPQ_RAT | chr1_164064815__1 | 0.64 | 0.93 | 0.87 | NO |
| sp\|P51400\|RED1_RAT | chr1_164064815__1 | 0.62 | 0.92 | 0.73 | NO |
| sp\|B2RYD2\|ESRP1_RAT | chr1_164064815__1 | 0.57 | 0.91 | 0.85 | NO |
| sp\|P13383\|NUCL_RAT | chr1_164064815__1 | 0.51 | 0.9 | 0.68 | NO |
| sp\|Q5XI28\|RAVR1_RAT | chr1_164064815__1 | 0.48 | 0.89 | 0.78 | NO |
| sp\|P68101\|IF2A_RAT | chr1_164064815__1 | 0.45 | 0.88 | 0.97 | NO |
